# Targeted phenolic profile of radler beers by HPLC-ESI-MS/MS: the added value of hesperidin to beer antioxidants

**DOI:** 10.1007/s13197-022-05536-8

**Published:** 2022-06-26

**Authors:** Paola Di Matteo, Martina Bortolami, Ludovica Di Virgilio, Rita Petrucci

**Affiliations:** Department of Basic and Applied Sciences for Engineering (SBAI), Sapienza University of Rome, Via del Castro Laurenziano, 7-00161 Rome, Italy

**Keywords:** Beverages, Citrus-beer, Hydroxybenzoic acids, Hydroxycinnamic acids, Flavonoids, HPLC-ESI-MS/MS-SIR

## Abstract

The well-known health beneficial properties of beer are mainly due to phenolic antioxidants. Citrus-flavored beers represent a growing side-market in the beer industry, sparingly investigated to date. The phenolic profile of commercial radler beers (R1, R2) was investigated to evaluate the impact of the lemon juice added to beer in the industrial production. Results were compared to those obtained for opportunely chosen commercial beer (B) and lemonade (L). The study was carried out by an HPLC-MS/MS with an electrospray ionization source in selected ion recording mode, analyzing in a single chromatographic run 26 compounds belonging to the different phenolic classes of hydroxybenzoic, hydroxycinnamic and caffeoylquinic acids, flavonoids and prenylflavonoids. Different phenolic profiles were found for R1 and R2, mainly ascribed to different malt/hop/recipe used for the beer. High to very high level of hesperidin were found in the radlers, so that a major impact on phenolic antioxidants of the radlers was due to the lemon. Similarly, a major impact of the lemon aromas was found, D-limonene being the dominant peak resulting from the GC-MS analysis of the volatile fraction of the radlers.

**Graphical Abstract:**

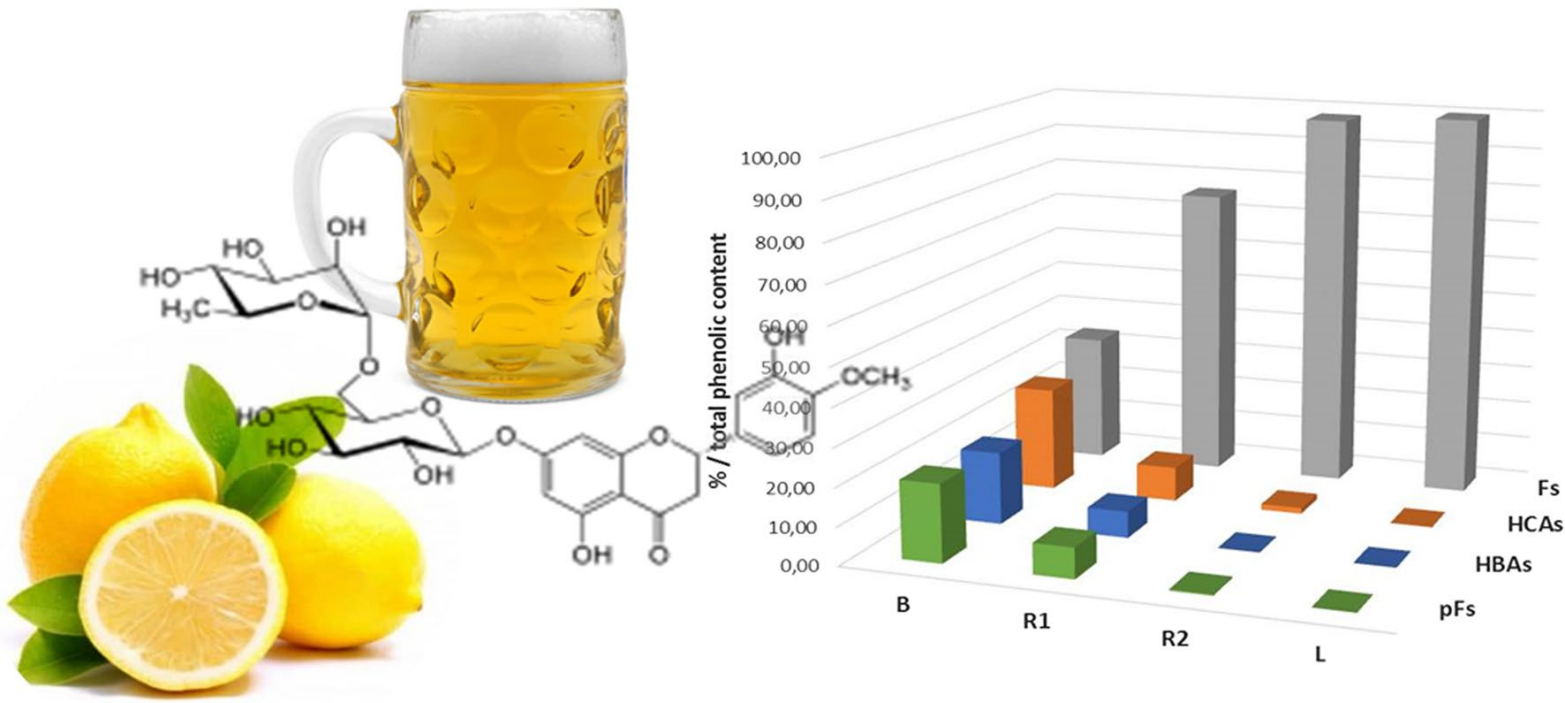

## Introduction

The beneficial effects exerted by the daily intake of citrus fruits are well-known and associated to the phytochemical composition of volatile and nonvolatile fractions, including terpene hydrocarbons, flavonoids and phenolic acids, with strong antioxidant and bioactive properties (Alu’datt et al. 2017; Shi et al. 2020; Singh et al. 2020; Mazzotti et al. 2021). The flavonoids composition of citrus fruits has been widely investigated, with distribution and content depending on citrus species, fruit part, fruit development and maturation (Gorinstein et al. 2001; Mazzotti et al. 2021; Mcharek and Hanchi 2017; Singh et al. 2020; Xi et al. 2017). *Citrus limon* (L.) Burm. is the most widespread *Citrus* species in the world after orange and mandarin, appreciated by consumers for its flavor and popular as health-promoting fruit (Xi et al. 2017). Among the bioactive flavonoids of lemon, high interest has been recently addressed to hesperidin, because of its high binding affinity to the cellular receptors of SARS-CoV-2 (Bellavite and Donzelli 2020; Santana et al. 2021).

Beer is a beverage largely consumed worldwide, and with coffee, tea, chocolate and wine, represents an important source for the daily intake of polyphenols, whose beneficial long-term effects on health have been largely supported (Rio et al. 2013; Reed and de Freitas 2020). Malt and hops are the main source of polyphenols in beer (Cortese et al. 2020; Gouvinhas et al. 2021); further, the effect of adding polyphenol-rich foods to beer has been recently evaluated (Trovato et al. 2021). Citrus-flavored beers represent a growing side-market in the beer industry, due to an increasing popularity among the consumers that appreciate the citrus fruits fresh-flavor on the beer aromas, the decreased alcohol content and a feeling of well-being on health. In particular, the radler is a beverage composed of beer and lemonade in equal parts, likely originated in Baviera at the beginning of the last century. Though a combined effect of phenolic content from beer and lemon might be expected, this beverage has been sparingly studied, at least up to our knowledge. The effect of the addition of the citrus flavors on the volatile and non-volatile profile of beer has been recently reported (Trovato et al. 2021). The phenolic composition of lemon has been also reported, mainly regarding the peel, rarely the juice, often in comparative studies with other citrus fruits, and in general phenolic compounds of different classes were investigated separately (Alu’datt et al. 2017; Gorinstein et al. 2001; Mazzotti et al. 2021; Mcharek and Hanchi 2017; Singh et al. 2020; Xi et al. 2017). Nowadays, mass spectrometry, coupled with chromatographic separation, is the most used technique for the analysis of complex matrices, suitable for clean, semi-purified or not pretreated samples (Cheiran et al. 2019; Chiarotto et al. 2019; Panusa et al. 2015; Petrucci et al. 2020a; Quifer-Rada et al. 2015).

The present study aimed to investigate the phenolic profile of the radler beverage and to evaluate the impact of the lemon juice on beer antioxidant fraction. For this purpose, two commercial Italian radlers, declared made with Italian malts and lemon juice, were analyzed by high performance liquid chromatography (HPLC) coupled with tandem mass spectrometry (MS/MS), with an electrospray ionization source (ESI) acquiring in selected ion recording (SIR) mode, by using a method previously developed for the identification and quantitation of fourteen phenolic compounds (Petrucci et al. 2020b), and up-gradated for the analysis of twelve phenolic compounds more. One Italian beer and one Italian lemonade, soft drink declared made with Italian lemon, were chosen and analyzed for a comparison.

The volatile fraction of the radler beers was also investigated by gas chromatography (GC) coupled with mass spectrometry (MS) and compared to those ones of beer and lemon juice, with the aim to obtain a comprehensive metabolic profile of the radler beer.

## Materials and methods

### Chemicals and reagents

3,4,5-trihydroxybenzoic acid (GA), 3,5-dihydroxybenzoic acid (3,5-DHBA), 3,4-dihydroxybenzoic acid (PCA), 5-caffeoylquinic acid (5CQA), 2,5-dihydroxybenzoic acid (2,5-DHBA), catechin (Cat), 4-caffeoylquinic acid (4CQA), *p*-hydroxybenzoic acid (pHBA), vanillic acid (VA), caffeic acid (CA), syringic acid (SyA), *m*-hydroxybenzoic acid (mHBA), 2,6-dihydroxybenzoic acid (2,6-DHBA), cumaric acid (CuA), sinapic acid (SA), ferulic acid (FA), rutin (Ru), myricitrin (My), quercetin-3-O-glucoside (Q3G), kampferol-3-O-rutinoside (K3R), salicylic acid (SaA), hesperidin (He), quercetin (Q), kampferol (K), isoxanthohumol (IsoX), xanthohumol (X), formic acid, and dichloromethane, were purchased from Sigma-Aldrich (Milano, Italy). HPLC-grade acetonitrile and methanol were Carlo Erba (Milano, Italy); HPLC-grade water was prepared with the Milli-Q purification system (Millipore, Vimodrone, Italy).

### Samples collection and preparation

Two Italian radlers (R1, R2), one Italian beer (B, same brand of R1) and one Italian lemonade soft drink (L) were purchased from a local supermarket and stored at 4 °C before use. Based on the label, Italian malts were used for B, R1 and R2. The alcohol content by volume (ABV) was 4.7% (B), 2% (R1) and 2% (R2), respectively. R1 contained concentrated lemon juice 3.2% (100% Italian lemon) and 42% beer; R2 contained concentrated lemon juice 2.7% (100% Italian lemon), 40% beer, orange, lime and acerola juice. L contained concentrate lemon juice 12% (100% Sicilian lemon). Data are resumed in Table 1.

**Table 1:** Analyzed commercial samples (beer B, radler beers R1 and R2, lemonade soft drink L), alcohol content by volume (ABV), labelled composition

Sample	Type	ABV	Labelled composition
B	Italian lager beer	4.7%	Italian malt
R1	Italian radler beer	2.0%	42% beer, Italian malt; concentrate lemon juice 3.2%, Italian lemon 100%
R2	Italian radler beer	2.0%	40% beer, Italian malt; concentrate lemon juice 2.7%, Italian lemon 100%; orange, lime, acerola juice
L	Italian lemonade	Soft drink	Concentrate lemon juice 12%, Sicilian lemon 100%

20 mL of each sample were degassed for 15 min in ultrasonic bath (Metason 60, Struers), filtered at 0.45 μm and stored at -20 °C. Prior to analysis, the samples were brought back to room temperature and appropriately diluted with the mobile phase (MilliQ water/acetonitrile, both formic acid 5 mM, 95:5 v/v).

### Standards solution preparation, calibration curves, quality-of-analysis parameters

A stock solution containing 3,5-DHBA, 2,5-DHBA, 2,6-DHBA, 4CQA, SaA, Cat, My, Q3G, K3R, He, IsoX and X was prepared by dissolving 1 mg/mL of each standard in methanol; working solutions were prepared by appropriate dilution with the mobile phase (A/B, 95:5, v/v, vide infra). The isomeric compounds were analyzed also separately to unambiguously assign the retention time. Calibration curves were calculated with equal-weighted least-squares linear regression analysis of the SIR peak area against the standard nominal concentration, by using 10, 20, 40, 60, and 80 µg/L solutions analyzed in triplicate (20 µL injected). Limit of detection (LOD) and quantitation (LOQ) were obtained as LOD = 3Sa/b and LOQ = 10Sa/b, respectively, where Sa and b are the estimated standard deviation and the slope of the analytical calibration function with a 95% confidence level, respectively (Trani et al. 2015). 40 µg/L solutions were used to evaluate intraday (five injections) and interday (triplicate injections in different days) precision, and results given as percent standard deviation (RSD%); 40 µg/L solutions were used to evaluate accuracy (triplicate injections), and results given as the percent difference between the nominal concentration and the measured one. The recovery test was carried out in duplicate by spiking five levels of concentration (10, 20, 40, 60, and 80 µg/L) into B diluted 1:100 with the mobile phase; the percent recovery was calculated as the ratio (peak area in B prepared by an additional method)/(peak area in solvent) and reported as RSD%. Matrix effect (ME) was evaluated as [(slope of the calibration curve in the matrix)/(slope of the calibration curve in the solvent) -1]x100. Linearity, sensitivity, precision, accuracy, recovery and ME are shown in Table 2.

**Table 2:** Quality-of-analysis parameters of the new 12 phenolic standards of the herein improved HPLC-ESI-MS/SIR method, previously developed (Petrucci et al. 2020b; Petrucci et al. 2021); chromatographic data (t_R_); mass spectral data ([M-H]^−^ m/z value); ^a^ triplicate analysis in three different days (40 µg/L); ^b^ five injections (40 µg/L); ^c^ triplicate analysis (40 µg/L); ^d^ five spikes (10, 20, 40, 60, 80 µg/L) in duplicate analysis

Standard	t_R_ (min)	R^2^	Calibration curve equation	LOD (µg/L)	LOQ (µg/L)	Interday^a^ (RSD%)	Intraday^b^ (RSD%)	Accuracy^c^ (%)	ME^d^	Recovery^d^ (RSD%)	[M-H]^−^ (m/z)
3,5-DHBA	4.36	1	y = 233.26 x + 714.82	0.070	0.22	5.12-4.28-1.20	5.11	+ 0.02	− 30	13.21	153
2,5-DHBA	6.67	0.9944	y = 181.64 x + 15.132	1.05	3.20	12.88-6.07-12.35	14.58	− 0.05	+ 41	10.02	153
Cat	6.95	0.9989	y = 87.602 x + 602.07	0.22	0.67	3.07-0.27-3.69	5.05	− 1.28	+ 22	20.66	289
4CQA	7.14	0.9956	y = 80.107 x – 231.87	0.92	2.80	4.37-3.96-3.29	7.58	− 0.033	+ 2	19.47	353
2,6-DHBA	10.53	0.9977	y = 1346.8 x + 11,852	5.02	15.21	3.35-1.20-0.62	8.61	+ 0.10	+ 16	16.41	153
My	16.29	0.9889	y = 61.696 x – 183.42	0.51	1.54	17.09-11.71-19.33	18.80	+ 1.28	− 40	20.76	463
Q3G	17.35	0.9818	y = 160.78 x – 785.84	1.70	5.15	8.84-4.45-18.32	11.91	− 5.68	− 25	24.51	463
K3R	19.57	0.9978	y = 120.56 x – 16.525	0.44	1.33	3.88-3.42-2.75	6.00	− 3.19	− 6	26.38	593
SaA	19.76	0.9961	y = 1207.8 x + 3745.3	10.23	31.01	0.89-0.90-0.35	2.68	− 0.55	− 7	11.75	137
He	23.22	0.9799	y = 58.793 x – 321.66	0.65	1.98	5.60-0.91-5.15	10.76	+ 0.80	− 12	11.00	609
IsoX	32.80	0.9935	y = 844.00 x – 1702.6	5.30	16.07	7.19-0.77-2.62	9.07	− 9.59	− 29	16.46	353
X	37.91	0.9715	y = 85.194 x + 4440.7	1.13	3.43	7.34-8.05-2.81	9.49	+ 6.27	+ 7	9.03	353

Calibration curves, LOD and LOQ of the other 14 standards (GA, PCA, pHBA, mHBA, 5CQA, VA, SyA, CA, FA, CuA, SA, Ru, Q, K) were used as previously reported in the literature (Petrucci et al. 2020b, 2021).

### HPLC-ESI-MS/MS instrumental conditions

A Waters 1525µ HPLC (Milford, MA) was used for the chromatographic separation, performed with a Waters XBridge C18 (150 × 2.1 mm i.d.) 5 μm analytical column; A (MilliQ water/formic acid 5 mM) and B (acetonitrile/formic acid 5 mM) were used as mobile phase for the elution binary gradient (Petrucci et al. 2020b) slightly modified. Briefly: 0–1 min, 5% B; 1–20 min, 16.5% B; 20–30 min, 40% B; 30–35 min, 60% B; 35–36 min, 80% B; 36–40 min, 80% B; 40–41 min, 5% B; 41–61 min, 5% B to equilibrate the column, flow rate of 0.20 mL/min. The Waters 996 photodiode array (PDA) detector was set for one spectrum/second, range 200–800 nm, resolution 1.2 nm. The Quattro Micro Tandem MS/MS with a Waters ESI source (Micromass, Manchester U.K.) acquired data in negative ionization ESI(-), capillary voltage 2.7 kV, cone voltage 27 V, source temperature 120 °C, desolvation temperature 350 °C, cone gas flow 40 L/h, desolvation gas flow 500 L/h (Petrucci et al. 2020b). 16 separated channels for 16 different *m/z* value for the selected ions [M–H]^−^ were used to acquire spectral data in SIR mode, dwell cell value of 0.200 s. Data acquisition, data handling, and instrument control were performed by MassLynx Software 4.1 v (Data Handling System for Windows, Micromass, U.K.).

### Stir bar sorptive extraction

Stir bar sorptive extraction was carried out by adding a polydimethylsiloxane coated stir bar (10 mm length, 3.2 mm o.d., 0.5 mm thickness, “Twister”, Gerstel, Germany) to 10 mL of the samples, in turn. After 1 h stirring at room temperature, the bar was removed, rinsed with MilliQ water, and placed in 350 µL of dichloromethane (Horák et al. 2007). After 1 h stirring, the extract was injected (1 µL) into the GC-MS for the analysis, in duplicate.

### GC-MS instrumental conditions

A GC-MS System Clarus 500 MS Turbo (PerkinElmer Instruments LLC, U.S.A.) was used to analyze the volatile fraction of the samples, with a Rtx®-1 capillary column (Restek, Bellefonte, U.S.A.), 60 m, 0.25 mm id, 0.25 μm df, and helium as carrier gas flowing at 1 mL/min, split ratio 1:10; injector temperature 250 °C, analysis program: 35 °C for 10 min, to 100 °C at 5 °C/min, to 201 °C at 3 °C/min, final temperature of 210 °C held for 40 min (Di Matteo G. et al. 2021; Di Matteo P. et al. 2021). The GC-MS interface temperature was 200 °C, 180 °C the source temperature. The acquisition was carried out in full scan mode, mass range 30 ÷ 200 Da, scan time 0.2 s. Data acquisition, data handling, and instrument control were performed by Turbomass 6.1.0 v PerkinElmer. Compounds were identified by comparison of mass spectra with NIST libraries.

### Statistical analysis

All samples were analyzed in triplicate and results reported in Table 3 as mean values ± standard deviation (SD). Data were analyzed by using the one-way analysis of variance (ANOVA). The significance of differences (p < 0.05) among samples was determined by the Tukey test.

**Table 3:** Phenolic compounds content in beer B, radler beers R1 and R2, lemonade L, as µg/L_beverage_ mean value ± SD from triplicate analysis by HPLC-ESI-MS/MS, SIR mode; nq = not quantitated; nd = not detected; ^a,b,c^ values with different letters are significantly different at *p* < 0.05

n.	Compound	t_R_ (min)	B (µg/L ± SD)	R1 (µg/L ± SD)	R2 (µg/L ± SD)	L (µg/L ± SD)	[M–H]^−^ (m/z)
1	3,4,5-trihydroxybenzoic acid	3.01	nd	nd	nd	nd	169
2	3,5-dihydroxybenzoic acid	4.36	nd	nd	nd	nd	153
3	3,4-dihydroxybenzoic acid	4.84	nd	nd	nd	nq	153
4	5-caffeoylquinic acid	6.56	nd	nd	nd	nd	353
5	2,5-dihydroxybenzoic acid	6.67	nd	nd	nd	nd	153
6	Catechin	7.05	1003.2 ± 73.9^a^	840.0 ± 58.8^a^	304.3 ± 14.21^b^	nd	289
7	4-caffeoylquinic acid	7.14	nd	nd	nd	nd	353
8	p-hydroxybenzoic acid	7.42	267.5 ± 41.4^a^	184.8 ± 33.7^a^	nd	nd	137
9	Vanillic acid	8.81	212.4 ± 26.5^a^	144.6 ± 2.6^b^	nq	nd	167
10	Caffeic acid	9.00	54.1 ± 0.30^a^	44.1 ± 2.0^b^	nd	nd	179
11	Syringic acid	9.20	107.2 ± 17.6^a^	112.1 ± 10.9^a^	nd	nd	197
12	m-hydroxybenzoic acid	10.22	nd	nd	nd	nd	137
13	2,6-dihydroxybenzoic acid	11.98	< 5.02	< 5.02	< 5.02	nd	153
14	Coumaric acid	13.45	194.2 ± 12.5^a^	121.1 ± 21.9^a^	94.0 ± 4.3^b^	< 60	163
15	Sinapic acid	15.48	201.0 ± 2.6^a^	136.0 ± 22.0^b^	93.5 ± 9.3^c^	159.8 ± 11.6^a^	223
16	Ferulic acid	15.54	448.8 ± 7.0^a^	311.7 ± 27.1^b^	213.2 ± 9.4^c^	< 60	193
17	Rutin	16.27	nd	< 90	< 90	211.6 ± 4.3	609
18	Myricitrin	15.96	nq	78.5 ± 25.7	nd	nq	463
19	Quercetin-3-O-glucoside	17.27	78.6 ± 3.8^a^	153.2 ± 19.5^b^	127.9 ± 8.8^b^	82.4 ± 10.3^c^	463
20	Kampferol-3-O-rutinoside	19.48	24.7 ± 0.5^a^	26.5 ± 9.4^a^	31.5 ± 8.0^a^	nd	593
21	Salicylic acid	19.86	39.0 ± 17^a^	28.7 ± 3.8^a^	nd	nd	137
22	Hesperidin	23.22	nd	3980.0 ± 610^a^	25,230 ± 140^b^	118,500 ± 13,500^c^	609
23	Quercetin	27.44	< 70	77.5 ± 2.6^a^	< 70	nd	301
24	Kampferol	30.01	< 60	< 60.0	nd	nd	285
25	Isoxanthohumol	32.70	60.7 ± 6.8^a^	84.1 ± 19.4^a^	103.6 ± 41.0^a^	nd	353
26	Xanthohumol	38.01	614.6 ± 129.6^a^	462.5 ± 254.3^a^	< 1.13	nd	353

## Results and discussion

### Phenolic profile by HPLC-ESI-MS/MS-SIR mode

26 phenolic compounds were investigated in two commercial radlers (R1 and R2, different brand), made with Italian malts and lemon juice, according to the label. Other citrus fruits besides lemon were labelled for R2. One beer (B, same brand of R1) and one lemonade soft drink (L), containing Sicilian lemon juice, according to the label, were analyzed for a comparison (Table 1).

10 Hydroxybenzoic acids (HBAs), 4 hydroxycinnamic acids (HCAs), 2 caffeoylquinic esters (CQAs), 8 flavonoids (Fs) and 2 prenylflavonoids (pFs) were included in the standards pool. In detail, numbered according to the elution order (t_R_, Table 3): 3,4,5-trihydroxybenzoic acid **1** (gallic acid, GA), 3,5-dihydroxybenzoic acid **2** (3,5-DHBA), 3,4-dihydroxybenzoic acid **3** (protocatecuic acid, PCA), 5-caffeoylquinic acid **4** (5CQA), 2,5-dihydroxybenzoic acid **5** (2,5-DHBA, gentisic acid), catechin **6** (Cat), 4-caffeoylquinic acid **7** (4CQA), *p*-hydroxybenzoic acid **8** (pHBA), vanillic acid **9** (VA), caffeic acid **10** (CA), syringic acid **11** (SyA), *m*-hydroxybenzoic acid **12** (mHBA), 2,6-dihydroxybenzoic acid **13** (2,6-DHBA), coumaric acid **14** (CuA), sinapic acid **15** (SA), ferulic acid **16** (FA), rutin **17** (Ru), myricitrin **18** (My), quercetin-3-O-glucoside **19** (Q3G), kampferol-3-O-rutinoside **20** (K3R), salicylic acid **21** (SaA), hesperidin **22** (He), quercetin **23** (Q), kampferol **24** (K), isoxanthohumol **25** (IsoX), xanthohumol **26** (X). The analysis was carried out by an HPLC-ESI-MS/MS in SIR mode method previously developed for 14 compounds (**1**, **3**, **4**, **8**–**12**, **14**–**17**, **23**, **24**) (Petrucci et al. 2020b, 2021) and herein slightly modified to include 12 more compounds (**2**, **5**–**7**, **13**, **18**–**22**, **25**, **26**), whose quality-of-analysis parameters are resumed in Table 2. Briefly, all calibration curves showed a good linearity in the investigated concentration range 10 ÷ 80 µg/L, as evidenced by the R^2^ values ranging within 0.9715 ÷ 1 (the minimum value 0.9715 was found for X), reported in Table 2; LOD and LOQ values were in the concentration ranges 0.070 ÷ 10.23 µg/L and 0.22 ÷ 31.01 µg/L, respectively (the higher LOD and LOQ values were found for SaA). Satisfactory data were obtained for accuracy (%, range − 9.59 ÷ 6.27) and precision (intraday, RSD% range 2.68 ÷ 18.80; interday, RSD% range 0.27 ÷ 19.33), the worst data found for My and 2,5-DHBA. The overall recovery percentages had RSD% in the range 9.03 ÷ 26.38, the worst values found for K3R and Q3G. ME varied between − 40 and + 41, but except My and 2,5-DHBA, a matrix effect from weak to medium (Zhang et al. 2019) was found for most of compounds. The improved method was confirmed suitable for fast analysis of complex matrices (Di Matteo P. et al., 2021).

Among the searched 26 compounds, 20 were identified in at least one sample and quantitated in most cases. Results, expressed as µg/L in the original sample, are reported in Table 3.

CQAs **4** and **7**, and the hydroxybenzoic acids (HBAs) **1**, **2**, **5** and **12** were not detected in any of the samples; conversely, HBAs **8**, **9**, **11** and **21** were identified and quantitated in B and R1, **13** was identified in B, R1 and R2, **3** was found in trace only in L. Therefore, the investigated HBAs seemed not to be characteristic components of the Sicilian lemon, used for L. Noteworthy, the phenolic acids profile of lemon has been sparingly reported and the few papers are aligned with our results (Alu’datt et al. 2017; Gorinstein et al. 2001; Singh et al. 2020; Xi et al. 2017). Some differences between R1 and R2 (different brand) are likely due to different starting materials (malts and/or hops) used for the beers (Cheiran et al. 2019; Cortese et al. 2020; Gouvinhas et al. 2021; Petrucci et al. 2020b, 2021). Conversely, similarity between B and R1 might be expected, since R1 is likely prepared with B (same brand): in fact, B and R1 were not significantly different (p < 0.05) for HBAs, except for VA **9**.

The hydroxycinnamic acids (HCAs) were identified in all samples, except CA **10**, found only in B and R1 (same brand). FA **16** was predominant in all alcoholic samples, as expected since it is typical of beer, while SA **15** was more abundant in L. R1 and R2 resulted significantly different (p < 0.05) for HCAs, R1 in general richer than R2, analogously to what observed for HBAs, described above. B and R1 resulted significantly different for HCAs, except for CuA **14**: the general decrease observed in R1 compared to B was likely due to the dilution step occurring during the radler production, that prevails over the possible intake from the lemon juice.

CuA, SA and FA (**14**–**16**) might be typical of the Italian lemon, used to prepare L, R1 and R2. Noteworthy, some authors reported the presence of HCAs **10** and **14**–**16** in fresh lemon, mainly in the peel (Gorinstein et al. 2001); others reported the presence of CA **10** in different fruit parts of lemon cultivars, juice included though in lower amounts, while very low content of FA **16** was found only in the peel, not in the juice (Xi et al. 2017).

Flavonoids were differently distributed among the analyzed samples.

Prenylflavonoids (pFs) from hops X **26** and IsoX **25** are characteristic of beer and were absent in L. Similar content of **25** was found in the alcoholic samples; conversely, a low quantity of **26** was found in R2, suggesting, once more, differences in the beer recipes used for R1 and R2, likely regarding hop, thermic treatments or pH (Zambrzycka-Szelewa et al. 2020).

Among flavonoids (Fs),Cat **6** was the most abundant in B, R1 and R2, in agreement with literature reporting **6** as the typical flavonoid of beer (Cheiran et al. 2019; Gouvinhas et al. 2021), and it was absent in L.

Q **23** and K **24** are typical of beer too (Di Matteo P. et al., 2021; Gouvinhas et al. 2021; Petrucci et al. 2020b, 2021; Quifer-Rada et al. 2015), mainly coming from hops. Quercetin was found in the free form (**23**) and in the bonded form, identified as Q3G **19**; conversely, kampferol was present mainly as the bonded form, identified as K3R **20**, though it was detected also in the free form (**24**) in B and R1. B, R1 and R2 resulted not significantly different (*p* < 0.05) with regard to **20**. Noteworthy, K3R **20** was reported as a discriminant compound for lager beer (Cheiran et al. 2019).

My **18** was found in trace in B and L, and it was quantitated in R1, in which the higher content was likely due to the intake from both B and the lemon juice. It was not detected in R2.

Conversely, Ru **17** and He **22** were found typical of the Italian lemon juice; particularly, He **22** was present in L in very high amount, in agreement with literature (Mcharek and Hanchi 2017; Singh et al. 2020; Xi et al. 2017). Ru **17** and He **22** were absent in B, consequentially their presence in R1 and R2 was due to the Italian lemon juice.

He **22**, Ru **17**, Q3G **19** and My **18** were found in L, with a content following the order **22** > > **17** > **19** > **18**. These data agree with literature reporting the biosynthesis and the accumulation of high level of flavonoid glycosides in citrus (Owens and McIntosh 2011). Free quercetin **23** was not detected in L, the same occurring for K **24** and its rutinoside derivative **20**. He **22** and Ru **17** were reported in the literature as the main representative flavonoids of lemon (Alu’datt et al. 2017; Mchareck and Hanchi, 2017; Xi et al. 2017).

Resuming, a different phenolic profile was observed for R1 and R2, mainly regarding the absence of HBAs in R2 and a different distribution of IsoX (**25**) and X (**26**). These results may be ascribed to differences in malt, hop, and brewing process for the production of the beers used to prepare R1 and R2. This is confirmed by the correlation found between R1 and B, same brand, that had substantially the same phenolic profile, except for hesperidin.

Conversely, R2 was found much richer than R1 in hesperidin **22**: since similar content of the concentrate Italian lemon juice is labelled for R1 and R2 (3.2% vs. 2.7%, respectively, see Table 1), such a higher content of He **22** in R2 was likely due to the presence of the other citrus fruits, orange and lime, reported on the label (see Table 1).

Summing up, the targeted phenolic profile of the Italian lemon juice has been firstly investigated, at least up to our knowledge, including HBAs, HCAs, Fs and pFs. Coumaric, sinapic and ferulic acids (**14**–**16**, respectively), rutin, myricitrin, quercetin-3-O-glucoside (**17**–**19**, respectively) and hesperidin **22**, were identified and quantitated where possible.

**Fig. 1:**
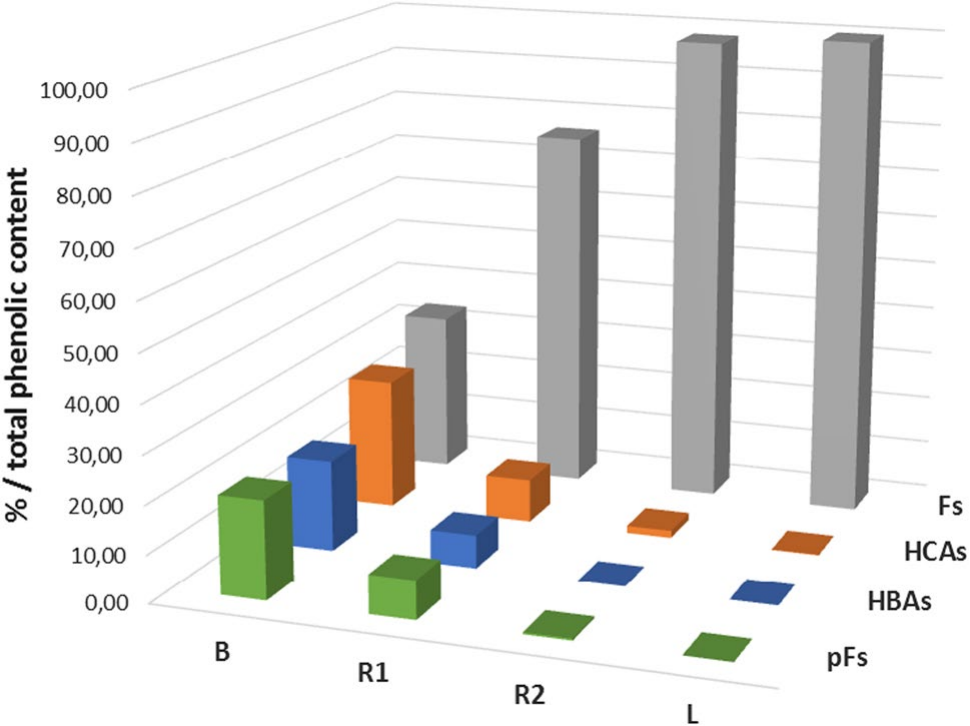
Comparative view of the distribution of each phenolic class in the analyzed beverages (B, R1, R2 and L), reported as percentage of each phenolic class respect to the total phenolic content calculated as the sum of all classes. Green: pFs, prenylflavonoids; blue: HBAs, hydroxybenzoic acids; brown: HCAs, hydroxycinnamic acids; grey: Fs, flavonoids

The impact of the lemon juice on the phenolic profile of beer was evaluated for the analyzed samples. The total content of the phenolic compounds of B, R1, R2 and L were summarized for classes (hydroxybenzoic acids HBAs, hydroxycinnamic acids HCAs, flavonoids Fs and prenylflavonoids pFs), and comparative results are plotted in Fig. 1. Radlers resulted strongly strengthened in antioxidants content respect to beer from both qualitative and quantitative aspects, with high level of hesperidin.

### GC-MS fingerprinting of the volatile fraction

The volatile fraction profile of R1, R2, B and L was investigated by GC-MS analysis (Di Matteo G. et al. 2021; Di Matteo P. et al. 2021) of the extracts obtained by a first stir bar extraction followed by a back-extraction in dichloromethane (Horák et al. 2007).

The untargeted analysis evidenced 23 peaks (**27**–**49** in Table 4), tentatively assigned by comparison of the fragmentation spectra with NIST libraries: 8 compounds resulted typical of B, 13 compounds resulted typical of L, and 2 compounds were found in both B and L. Most of the 23 compounds were found in the volatile profile of R1 and R2.

**Table 4:** Compounds tentatively identified in the volatile fraction of B, R1, R2 and L, by GC-MS analysis and comparision of fragmentation spectra with NIST libraries. d: detected; nd: not detected

n.	Compound	t_R_ (min)	B	R1	R2	L
**27**	Heptane	9.48	d	d	d	d
**28**	Isoamyl alchohol	10.71	d	d	d	nd
**29**	Diacetone alchohol	15.85	nd	d	d	d
**30**	*m*-xylene	17.94	d	d	d	d
**31**	Isoamyl acetate	18.22	d	d	d	nd
**32**	Lsoamyl n-eptanoate	18.34	d	d	d	nd
**33**	Ethyl caproate	23.60	d	d	d	nd
**34**	α-pinene	23.63	nd	nd	nd	d
**35**	Isocineole	24.55	nd	d	d	d
**36**	β-cimene	24.85	nd	d	d	d
**37**	D-limonene	25.25	nd	d	d	d
**38**	γ-terpinene	26.41	nd	d	d	d
**39**	(+)-4-carene	27.67	nd	d	d	d
**40**	N-hydroxymethyl-2-phenylacetamide	27.87	d	nd	nd	nd
**41**	β-fenchol	28.53	nd	d	d	d
**42**	4-amino-1-pentanol	28.64	d	nd	nd	nd
**43**	Neodihydro carveol	29.66	nd	d	d	d
**44**	Cosmene	30.47	nd	nd	nd	d
**45**	Vinyl-o-xylene	31.05	nd	nd	d	d
**46**	Terpinen-4-ol	31.15	nd	d	d	d
**47**	α-terpineol	31.58	nd	d	d	d
**48**	Ethyl caprylate	31.78	d	d	d	nd
**49**	Phenylethyl acetate	33.78	d	d	d	nd

Peaks evidenced in B were assigned to isoamyl alchohol **28**, isoamyl acetate **31**, ethyl caproate **33**, ethyl caprylate **48**, and phenylethyl acetate **49**, in agreement with literature, reporting them generally present in lager beers (Di Matteo P. et al., 2021; Horák et al. 2007; Nešpor et al. 2019); further, isoamyl *n*-eptanoate **32**, *N*-hydroxymethyl-2-phenylacetamide **40** and 4-amino-1-pentanol **42** were not previously reported, up to our knowledge. All compounds were confirmed in R1 and R2, except **40** and **42**.

Peaks evidenced in L were assigned to β-cimene **36**, D-limonene **37**, γ-terpinene **38**, β-fenchol **41**, terpinen-4-ol **46**, and α-terpineol **47**, in agreement with literature, reporting them in Italian lemon (Giuffrè et al. 2019; Scurria et al. 2021; Trovato et al. 2021); further, diacetone alchohol **29**, α-pinene **34**, isocineole **35**, 4-carene **39**, neodihydro carveol **43**, cosmene **44** and vinyl-o-xylene **45** were tentatively assigned. All compounds were confirmed in R1 and R2, except for α-pinene **34** and cosmene **44**, and vinyl-*o*-xylene **45**, detected in R2 but not in R1.

Heptane **27** and *m*-xylene **30** were found in all the samples.

**Fig. 2:**
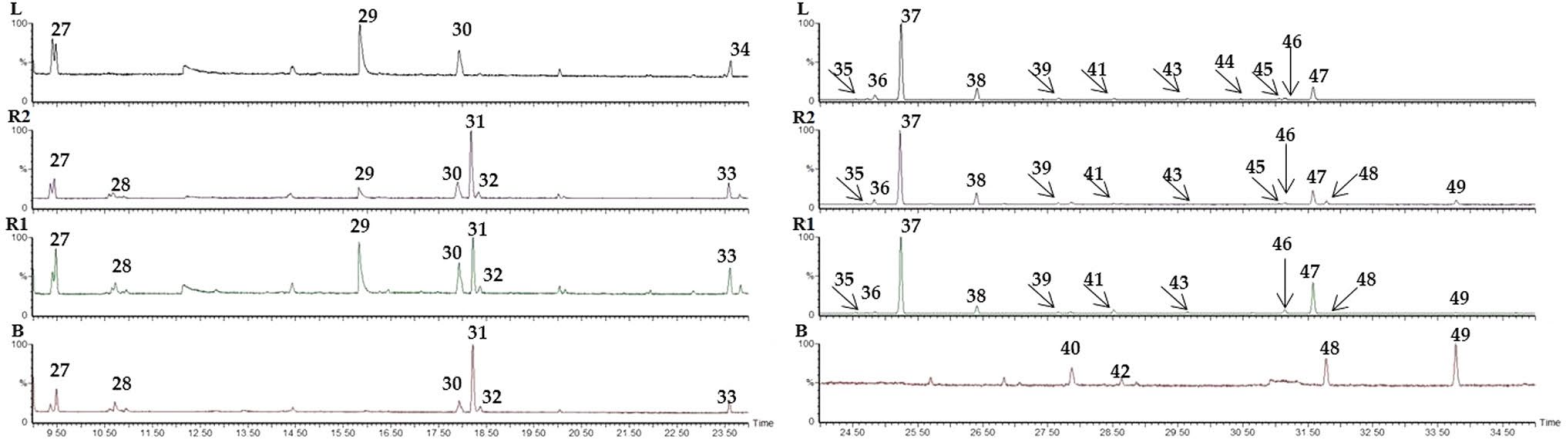
GC-MS volatile fraction profile of B, R1, R2 and L, from bottom to top, respectively

A major impact of lemon aromas was found on the radlers, D-limonene being the dominant peak, as shown in the total ion chromatograms (TIC) in Fig. 2. Qualitative data are resumed in Table 4.

## Conclusion

The present work aimed to characterize the phenolic fraction of radler beer, beverage composed of equal parts of beer and lemonade, growing in popularity because of the citrus fruits fresh-flavor on the beer aromas, the decreased alcohol content and a feeling of well-being on health. For this purpose, two independent commercial radlers, composed of lager beers made with Italian malts and Italian lemons, were analyzed by liquid chromatography (HPLC) coupled with a tandem mass spectrometer (MS/MS) working in Selected Ion Recording (SIR) mass spectrometry technique, by using a modified method previously developed. The validation parameters of the herein up-gradated method are given. One lager beer and one lemonade, made with Italian malts and Italian lemons, respectively, were also investigated for a comparison. From the targeted analysis of 26 phenolic compounds including 10 hydroxybenzoic acids (HBAs), 6 hydroxycinnamic acids and derivatives (HCAs), 8 flavonoids (Fs) and 2 prenylflavonoids (pFs), 20 compounds were identified in at least one of the analyzed sample, and quantitated in most cases. Noteworthy, the phenolic profile of the Italian lemon juice has been firstly investigated, at least up to our knowledge, including HBAs, HCAs, Fs and pFs: coumaric, sinapic and ferulic acids (**14**–**16**, respectively), rutin, myricitrin, quercetin-3-O-glucoside (**17**–**19**, respectively) and hesperidin **22**, were identified and quantitated where possible. A very high content of hesperidin was found in the lemonade, as expected because it is typical of citrus fruits.

Some differences were observed between the two samples of radlers, mainly regarding hydroxybenzoic acids, hydroxycinnamic acids and prenyflavonoids, that means the typical phenolic compounds of beer: such differences might be due to malt, hop and/or brewing process used to produce the beers for the radlers. Since sinapic acid, rutin and quercetin-3-*O*-glucoside, besides coumaric and ferulic acids at a very lesser extent, were found also in the lemonade, their content in the radlers might be due to both beer and lemon. High to very high level of hesperidin were found in the radlers, so that a major impact on phenolic antioxidants of the radlers was due to the lemon. A major impact of the lemon aromas was also found, D-limonene being the dominant peak resulting from the GC-MS analysis of the volatile fraction of the radlers. Besides the beneficial antioxidants of beer, the radler resulted strongly enriched by the citrus fruits flavonoids, mainly hesperidin, and by lemon aromas as D-limonene.

## Data Availability

Data are available on request from corresponding author.
